# Unplanned intensive care unit readmission after surgical treatment in patients with newly diagnosed glioblastoma — forfeiture of surgically achieved advantages?

**DOI:** 10.1007/s10143-022-01938-6

**Published:** 2023-01-03

**Authors:** Felix Lehmann, Anna-Laura Potthoff, Valeri Borger, Muriel Heimann, Stefan Felix Ehrentraut, Christina Schaub, Christian Putensen, Johannes Weller, Christian Bode, Hartmut Vatter, Ulrich Herrlinger, Patrick Schuss, Niklas Schäfer, Matthias Schneider

**Affiliations:** 1 Department of Anesthesiology and Intensive Care Medicine, University Hospital Bonn, Venusberg-Campus 1, 53127 Bonn, Germany; 2 Department of Neurosurgery, University Hospital Bonn, Bonn, Germany; 3 Division of Clinical Neuro-Oncology, Department of Neurology, University Hospital Bonn, Bonn, Germany; 4 Department of Neurosurgery, BG Klinikum Unfallkrankenhaus Berlin gGmbH, Berlin, Germany

**Keywords:** Critical care, Glioblastoma, Surgery, Dexamethasone

## Abstract

Postoperative intensive care unit (ICU) monitoring is an established option to ensure patient safety after resection of newly diagnosed glioblastoma. In contrast, secondary unplanned ICU readmission following complicating events during the initial postoperative course might be associated with severe morbidity and impair initially intended surgical benefit. In the present study, we assessed the prognostic impact of secondary ICU readmission and aimed to identify preoperatively ascertainable risk factors for the development of such adverse events in patients treated surgically for newly diagnosed glioblastoma. Between 2013 and 2018, 240 patients were surgically treated for newly diagnosed glioblastoma at the authors’ neuro-oncological center. Secondary ICU readmission was defined as any unplanned admission to the ICU during initial hospital stay. A multivariable logistic regression analysis was performed to identify preoperatively measurable risk factors for unplanned ICU readmission. Nineteen of 240 glioblastoma patients (8%) were readmitted to the ICU. Median overall survival of patients with unplanned ICU readmission was 9 months compared to 17 months for patients without secondary ICU readmission (*p*=0.008). Multivariable analysis identified “preoperative administration of dexamethasone > 7 days” (*p*=0.002) as a significant and independent predictor of secondary unplanned ICU admission. Secondary ICU readmission following surgery for newly diagnosed glioblastoma is significantly associated with poor survival and thus may negate surgically achieved prerequisites for further treatment. This underlines the indispensability of precise patient selection as well as the importance of further scientific debate on these highly relevant aspects for patient safety.

## Introduction

The treatment of patients with newly diagnosed glioblastoma follows an established therapeutic regime [1]. The initial phase is the timely diagnosis, including the determination of the molecular pathology within the framework of a surgical treatment [1]. Maximal safe resection thereby constitutes a key element in the subsequent therapeutic sequence [2–5]. Due to continuous improvements in surgical techniques and perioperative optimization, increasingly complex surgical procedures are becoming feasible in more comorbid patients [6, 7]. Preoperative refinement along with the identification of risk factors is essential in this context to ensure effective surgical treatment of affected (high-risk) patients [8, 9]. Rapid recovery of the patient after surgical treatment is crucial for a prompt subsequent individualized adjuvant therapy (radio- and/or chemotherapy) [10–12]. Since adjuvant therapy with its debilitating effects requires an appropriate constitution of the patient, efforts to identify risk factors that could delay timely therapy (e.g., prolonged mechanical ventilation) have increased recently [13]. An often neglected issue is the unplanned intensive care unit (ICU) readmission of patients with glioblastoma. Such an unplanned ICU readmission might represent a surrogate parameter for a variety of potential underlying problems (e.g., postoperative complications, internal medicine complications, epileptic events).

Given the limited amount of literature on this issue regarding glioblastoma patients, we investigated the likelihood, the reasons, and potential preoperative identifiable risk factors for unplanned ICU readmission in patients undergoing surgery for newly diagnosed glioblastoma.

## Methods

### Patients

Between 2013 and 2018, 240 patients with newly diagnosed glioblastoma underwent neurosurgical treatment at the Neuro-Oncology Center of the University Hospital Bonn. Merely patients with histopathological proven glioblastoma were considered in the following analyses. The present study was approved by the local ethics committee of the University Hospital Bonn. Thereafter, preoperatively identifiable characteristics from the patients to be included were transferred to a computer-based database (SPSS, version 26, IBM Corp., Armonk, NY) for further analysis.

Preoperative available information included patient age, body mass index (BMI), comorbidities, and neurological status, as well as information on potential preoperative tumor-associated epilepsy (TAE), tumor volume, and preoperative administration of glucocorticoids. The patient’s functional status prior to surgery was assessed using the Karnofsky Performance Scale (KPS), dichotomizing patients into two groups (KPS < 70 versus KPS ≥ 70). The comorbidity burden of the individual patient was measured using the Charlson comorbidity index (CCI) with incorporation of age [14]. Preoperative administration of dexamethasone ≥ 7 days was defined as prolonged cerebral edema therapy.

All procedures were conducted by or under the supervision of board-certified neurosurgeons. Patients with suspected high-grade gliomas on the basis of neuroimaging received 5-aminolevulinic acid preoperatively, facilitating fluorescence-guided tumor resection. Intraoperative neuronavigation was usually implemented to tailor the craniotomy and/or aid the surgeon in estimating the extent of resection. In addition, multimodal intraoperative neuromonitoring was performed for tumors nearby suspected eloquent brain functional areas.

For all procedures, immediate extubation was routinely intended after the surgical procedure. Postoperative clinical monitoring was performed in a dedicated neurosurgical intermediate care or intensive care unit (IMCU/ICU) until the next day. Patients with uneventful postoperative monitoring were then discharged to the normal ward.

Unplanned ICU readmission was defined as admission of a patient who had already been admitted to the ICU once during the same hospital stay.

### Statistics

Fisher’s exact test was utilized to analyze unpaired categorical and binary variables in contingency tables. Comparison of continuous variables was performed using Mann–Whitney *U* test since the data were mostly not normally distributed. OS was analyzed with the Kaplan–Meier method using the Gehan–Breslow–Wilcoxon test. Results with *p* < 0.05 were considered statistically significant. In addition, a stepwise backward method was used to develop a multivariable logistic regression model in order to find independent as well as preoperatively identifiable clinical predictors for unplanned postoperative ICU readmission in patients with surgically treated glioblastoma.

## Results

### Patient characteristics

During the period from 2013 to 2018, a total of 240 patients underwent surgical treatment for newly diagnosed glioblastoma at the Neuro-Oncology Center of the University Hospital Bonn and were included in further analysis.

Nineteen of 240 patients with surgically treated glioblastoma (8%) were readmitted to ICU after they were deemed eligible for transfer to normal wards due to their uneventful initial postoperative course. Details regarding distinct preoperative elicitable parameters among patients with and without unplanned ICU readmission are listed in Table 1.

**Table 1:** Preoperative identifiable risk factors for unplanned ICU readmission in patients with newly diagnosed glioblastoma

	**Patients w/o unplanned ICU readmission (*n*=211)**	**Patients with unplanned ICU readmission (*n*=19)**	***p*-value**
Median age (IQR; yrs)	64 (54–72)	59 (53–76)	0.9
Preoperative KPS ≥ 70	208 (94%)	19 (100%)	0.61
Preoperative tumor-associated epilepsy	67 (30%)	5 (26%)	0.8
Tumor volume (IQR, ml)	34 (12–72)	51 (13–88)	0.49
BMI > 30	42 (19%)	1 (5%)	0.21
Age-adjusted CCI ≥ 5	38 (17%)	4 (21%)	0.75
Prolonged preoperative dexamethasone medication (> 7 days)	60 (27%)	12 (63%)	0.003
ASA ≥ 3	59 (27%)	7 (39%)	0.28
30 day mortality	4 (2%)	4 (21%)	0.02
Median OS (IQR; mo)	17 (10–25)	9 (2–21)	0.008

*IQR*, interquartile range, *yrs*, years, *KPS*, Karnofsky Performance Scale, *BMI*, body mass index, *CCI*, Charlson comorbidity index, *ASA*, American Society of Anesthesiology, *OS*, overall survival, *mo*, months

### Reasons for unplanned ICU readmission

Reasons for unplanned postoperative ICU readmission in the aforementioned 19 patients with surgically treated glioblastoma (8%) were noted in the medical records to be as follows: local/epidural/subdural bleeding (8/19, 42%), neurologic deterioration (6/19, 32%), respiratory failure (3/19, 16%), cardiovascular instability (1/19, 5%), and other complications (1/19, 5%). The 3 cases of respiratory failure counted for pulmonary embolism in 2 cases (22%) and exacerbation of postoperatively new onset pneumonia on day 4 following surgery in 1 case (5%). Cardiovascular instability in 1 patient was due to early postoperative infarction at day 3 after surgery with the need for endovascular stenting. The one case listed as other complications featured an intestinal perforation as an independent complication that resulted in an unplanned ICU readmission.

### Tumor-/patient-related risk factors

Regarding preoperative risk factors, there was no significant difference in tumor volume between patients with and without unplanned ICU readmission after surgically resected glioblastoma. Patients with an uneventful postoperative course exhibited a median tumor volume of 34 ml (IQR 12–72) while patients with unplanned ICU readmission presented with a larger median tumor volume of 51 ml (IQR 13–88), but without reaching significance (*p*=0.49; Table 1). Regarding the potential impact of comorbidity burden, 21% of patients with unplanned ICU readmission were more severely affected (age-adjusted CCI ≥ 5) compared to about 17% of patients with unremarkable postoperative course, yet no statistically significant difference was detected (*p*=0.75; Table 1). MGMT promoter methylation status did not significantly differ between the groups with and without secondary ICU readmission: 8 of 19 patients (42%) with unplanned ICU readmission revealed hypermethylated status compared to 87 of 211 patients (41%) without unplanned ICU readmission (*p*=1.0).

The preoperative median white blood cell count (WBC) was 10.6 G/l (IQR 7.2–14.5) in patients with an unremarkable postoperative course. Patients with unplanned ICU readmission presented with a preoperative median WBC of 15.5 G/l (IQR 8.7–19.1). This difference was statistically significant (*p*=0.027). Similarly, preoperative prolonged administration of dexamethasone (>7 days) was significantly more frequent in patients with unplanned ICU readmission compared to patients without secondary ICU readmission (63% versus 27%, *p*=0.003, *OR* 4.6, 95% *CI* 1.7–12.2, Table 1).

### Implications of the unplanned ICU readmission on overall survival

**Fig. 1:**
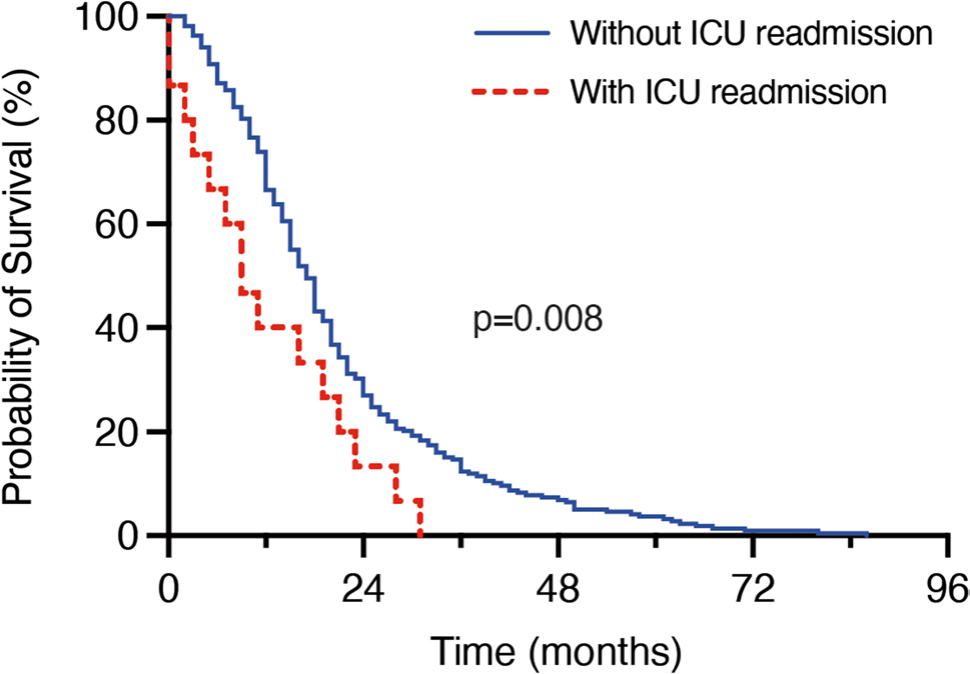
A Kaplan–Meier curve for association of unplanned ICU readmission and OS. ICU, intensive care unit; OS, overall survival

Patients with neurosurgically treated glioblastoma demonstrated significantly worse mOS when unplanned ICU readmission occurred postoperatively. Patients with an unaffected postoperative course achieved an mOS of 17 months (95% *CI* 15.4–18.6), whereas patients with an unplanned ICU readmission had an mOS of 9 months (95% *CI* 6.2–11.8; *p*=0.008; Fig. 1).

Multivariable analysis under consideration of age, preoperative KPS, preoperative CCI, MGMT promoter methylation status, IDH status and occurrence of ICU readmission, identified “un-methylated MGMT status” (*p*=0.001, *OR* 2.7, 95% *CI* 1.5–5.0), “age >65 years” (*p*<0.001, *OR* 6.3, 95% *CI* 3.9–11.6) as well as “occurrence of ICU readmission” (*p*=0.006, *OR* 6.0, 95% *CI* 1.7–21.8) as significant and independent predictors for worsened OS (Nagelkerke’s *R*^2^=0.281).

### Multivariable analysis

The multivariable regression analysis revealed “preoperative prolonged administration of dexamethasone” as the only significant, independent, and preoperatively identifiable predictor for an unplanned postoperative ICU readmission in patients with surgical resection for newly diagnosed glioblastoma (*p*=0.002, *OR* 4.6, 95% *CI* 1.7–12.2; Nagelkerke’s *R*^2^=0.094).

## Discussion

Despite longstanding versatile endeavors in treatment optimization, the diagnosis of glioblastoma is still afflicted with a dismal prognosis. The fact that the slightest deviation from the standardized treatment protocol (e.g., prolonged mechanical ventilation) might result in a significant loss of lifetime/quality of life is therefore gaining attention [13]. Thus, it is pertinent to consider not only potential preoperative risk factors but also (adverse) treatment effects in order to be able to adapt clinical assessment/counseling/management of affected patients as well as their relatives [15, 9, 7, 16, 8, 17].

Under the impression of possible complications of an ICU admission, the repelling impression of an “apparatus medicine” as well as the increasing financial/capacity pressure, an increasing avoidance of postoperative ICU monitoring has recently gained interest [18–20]. However, the (desired) reduction of postoperative ICU monitoring mandates a detailed and individualized consideration of the patient’s risk/benefit profile in order to weigh the safety of postoperative monitoring against unnecessary ICU treatment [21]. An important parameter for assessing the need for postoperative ICU monitoring is an unplanned ICU readmission. Therefore, the present study intended to provide insight into the risk profile of patients with glioblastoma who were cleared for transfer from the ICU by an interdisciplinary team of intensivists after an uneventful postoperative course — who then had to receive unplanned intensive medical care again.

After previous studies identified higher comorbidity burden as an indicator for the need for postoperative ICU monitoring after elective craniotomy, the present data indicate that increased comorbidity burden (as measured by CCI) does not appear to contribute to, at least, a significantly increased rate of unplanned ICU readmission [19]. Unfortunately, current guidelines do not allow to define precise standards for ICU requirements after elective craniotomy for brain tumors; at most, an individual assessment based on possible neurological deficits is advisable [22]. In the present study, preoperative intake of dexamethasone > 7 days was significantly associated with an elevated risk of secondary ICU readmission. Dexamethasone is commonly used to reduce peritumoral edema-related symptoms in several neurooncological diseases [23]. Dexamethasone is hypothesized to exert edema-reducing effects through improvement of the blood–brain-barrier functioning via upregulation of tight-junction proteins and inhibition of inflammatory signaling pathways resulting in decreased vessel permeability [23–26]. Though dexamethasone is quite effective in the reduction of vasogenic edematous volumes, a wide range of systemic adverse effects like hyperglycemia, cushingoid appearance, and psychiatric alterations among many others is reported to reach up to about 50% of treated patients [27, 28]. Dexamethasone is known to induce heart rate and blood pressure elevation as well as to elevate plasma cholesterol and triglycerides among others [29, 30]. Glucocorticoids have been shown to be associated with an elevated risk of left ventricular free wall rupture by delaying myocardial scar formation following acute myocardial infarction [30]. Moreover, patients treated with glucocorticoids are at a higher risk of venous thrombembolism [31] and pulmonary embolism [32]. These effects may predispose patients to coronary heart disease and elevated risk profiles following surgical interventions especially in case of high doses and prolonged dexamethasone intake. Though the risk of secondary hemorrhage in cranial surgery dependent on the use of dexamethasone has not intensively been studied and clear evidence of a beneficial or negative effect of dexamethasone is still lacking, so far [33], there are reports pointing at a potential association of glucocorticoid intake and postoperative hemorrhage. In a real-world practice setting with 36 US children’s hospitals, retrospective analysis of 139,715 patients that had undergone surgery for tonsillectomy revealed dexamethasone use to be associated with an absolute increased risk of revisits for bleeding [34]. Side effects involving the central nervous system mostly affect psychiatric and cognitive disturbances [35]. Besides to an incidence of depression of 40.5% of patients with corticosteroid intake, corticosteroid-related psychosis and delirium are reported to occur with an incidence of about 14% and 10% of treated patients, respectively [36]. Neuroimaging studies have shown corticosteroid use to result in a decrease of hippocampal volume as well as brain atrophy due to decreased blood flow in distinct brain areas resulting in consciousness disorders, memory deficits, and delirium [37, 36]. There is growing literature implicating dexamethasone administration to result in worsened overall prognosis in glioblastoma patients. Shields et al. reported a significantly reduced OS of 13 months for dexamethasone use with concurrent radiotherapy compared to 23 months for radiotherapy without dexamethasone (*p*<0.0001) [38]. Similarly, in a retrospective analysis of > 2000 glioblastoma patients, corticosteroid use at the start of radiotherapy without or with temozolomide was associated with poor prognosis (OS of 12 months with dexamethasone versus (vs) 17 months without dexamethasone, *p*=0.001) [39]. Wong and colleagues reported reduced OS in case of dexamethasone administration in patients with recurrent glioblastoma receiving either tumor-treating fields (TTFields) or chemotherapy (5 vs 11 months in the TTFields cohort, *p*=0.0001; 6 months vs 9 months in the chemotherapy cohort, *p*=0.0009) [40]. The present study is the first to link preoperative dexamethasone use to unplanned secondary admission to the ICU following severe early postoperative unfavorable events. In view of the growing literature indicating a negative prognostic influence of dexamethasone in glioblastoma disease, future endeavors might not only comprehensively delineate the impact of dexamethasone administration in glioblastoma patients but also point out alternative treatment options for tumor-induced edema management. A combinatorial pharmacological blockade of vascular endothelial growth factor (VEGF) and angiopoietin-2 — the latter of which enhances vascular permeability via receptor tyrosine kinase modulation in vascular endothelial cells — are of current interest [41, 42]. Against this backdrop, clinical trials on alternative substances with the potential to reduce peritumoral edema are highly warranted. The present data may provide a basis for the initiation of multicenter registries and further studies to comprehensively investigate the risk factors as well as the overall impact of unplanned ICU readmission in glioblastoma surgery.

### Limitations

The present study has several limitations. Data collection was performed retrospectively and patients were not randomized, but treated according to the preferences of the treating physicians. Furthermore, the group of 19 patients with unplanned ICU readmission was quite small and therefore hardly allowed any valid conclusions to be drawn about the underlying causes.

## Conclusions

Secondary ICU readmission following surgery for primary glioblastoma is significantly related to poor OS. The present data indicate prolonged preoperative dexamethasone use to be associated with an elevated risk for secondary readmission in the early postoperative course. Since secondary ICU readmission may obliterate the previously surgically achieved premises for further treatment in patients with newly diagnosed glioblastoma, continued scientific engagement with this highly relevant issue for patient safety remains mandatory.

### Availability of data and material

Restrictions apply to the availability of these data due to privacy restrictions.
